# Epidemiology and pathogenesis of stroke in preterm infants: A systematic review

**DOI:** 10.3233/NPM-200597

**Published:** 2022-01-27

**Authors:** B. Roy, K. Walker, C. Morgan, M. Finch-Edmondson, C. Galea, M. Epi, N. Badawi, I. Novak

**Affiliations:** a Discipline of Child and Adolescent Health, Faculty of Medicine and Health, The University of Sydney, Sydney, NSW, Australia; b School of Medicine, The University of Notre Dame Australia, Sydney, NSW, Australia; c The Mater Hospital, Sydney, NSW, Australia; d The George Institute for Global Health, Sydney, NSW, Australia; e Newborn Care, Royal Prince Alfred Hospital, Sydney, NSW, Australia; f Cerebral Palsy Alliance Research Institute, Sydney, NSW, Australia; g Grace Centre for Newborn Intensive Care, Sydney Children’s Hospital Network, Westmead, NSW, Australia

**Keywords:** Arterial ischemic stroke, cerebral sinovenous thrombosis, periventricular hemorrhagic infarction, neonatal stroke, perinatal stroke, stroke in preterm infants

## Abstract

**BACKGROUND:**

Perinatal stroke is one of the principal causes of cerebral palsy (CP) in preterm infants. Stroke in preterm infants is different from stroke in term infants, given the differences in brain maturation and the mechanisms of injury exclusive to the immature brain. We conducted a systematic review to explore the epidemiology and pathogenesis of periventricular hemorrhagic infarction (PVHI), perinatal arterial ischemic stroke (PAIS) and cerebral sinovenous thrombosis (CSVT) in preterm infants.

**METHODS:**

Studies were identified based on predefined study criteria from MEDLINE, EMBASE, SCOPUS and WEB OF SCIENCE electronic databases from 2000 –2019. Results were combined using descriptive statistics.

**RESULTS:**

Fourteen studies encompassed 546 stroke cases in preterm infants between 23 –36 weeks gestational ages and birth weights between 450 –3500 grams. Eighty percent (436/546) of the stroke cases were PVHI, 17%(93/546) were PAIS and 3%(17/546) were CSVT. Parietal PVHI was more common than temporal and frontal lobe PVHI. For PAIS, left middle cerebral artery (MCA) was more common than right MCA or cerebellar stroke. For CSVT partial or complete thrombosis in the transverse sinus was universal. All cases included multiple possible risk factors, but the data were discordant precluding aggregation within a meta-analysis.

**CONCLUSION:**

This systematic review confirms paucity of data regarding the etiology and the precise causal pathway of stroke in preterm infants. Moreover, the preterm infants unlike the term infants do not typically present with seizures. Hence high index of clinical suspicion and routine cUS will assist in the timely diagnosis and understanding of stroke in this population.

## 1 Introduction

Stroke in preterm infants occurs from the 20th to 37th gestational week. Stroke may happen either before birth (known as fetal or in-utero stroke) or after birth, up to 37 weeks postmenstrual age [1]. Long-term morbidities include motor impairment, cognitive and speech disorders, developmental delay, cerebral palsy and/or epilepsy [2, 3]. The estimated prevalence of stroke in preterm infants varies widely in published literature; ranging from 1:1600 to 1:8000 [4, 5]. Stroke has been more commonly studied in term infants, therefore the global burden of perinatal stroke in preterm infants is undetermined. These gaps in knowledge are due to paucity of published data and small single centre studies [6, 7].

In literature, the common types of stroke described in preterm infants are periventricular hemorrhagic infarction (PVHI), perinatal arterial ischemic stroke (PAIS) and cerebral sinovenous thrombosis (CSVT). PVHI is a periventricular venous congestion due to the obstruction of the terminal vein and impaired blood flow in the medullary vein following germinal matrix hemorrhage. PAIS is disruption of blood flow to the cerebral artery due to thrombosis, embolism, vasospasm or hypoxic-ischemic encephalopathy. CSVT is a clot in the venous sinuses of the brain causing blockade of the blood draining from the brain. This causes blood cells to break and leak into the brain tissue causing hemorrhage.

The diagnosis of stroke is established from neuroradiological investigations [8]. Neuroimaging is routinely ordered in full-term infants presenting with seizures or unexplained apneas. While, in preterm infants, symptoms like apneas can also be linked to problems of prematurity rather than to stroke alone. In addition, the pathophysiology of stroke in preterm infants is different to the other brain injuries that are common to preterm infants like intraventricular hemorrhage (IVH) and periventricular leukomalacia (PVL) [9].

Literature suggests the likely causal pathway of stroke in preterm infants involves a multiplicity of risk factors rather than any single risk factor. Some of the known independent risk factors for PAIS include: Fetal heart rate abnormality (*p* = 0.008), neonatal hypoglycemia (*p* = 0.02), twin to twin transfusion syndrome (*p* = 0.005), dehydration, infection, patent ductus arteriosus and prothrombotic disorders [10, 11]. The presence of more than three potential risk factors is known to increase the risk of stroke more than any of these single factors in isolation: Maternal age, race, infertility, miscarriage, intrauterine growth restriction and gestational diabetes [11]. In the scant literature available, placental abnormalities such as placental weight < 10th percentile (*p* = 0.00), placental infarction (*p* = 0.00), malperfusion (*p* = 0.01), and chorioamnionitis (*p* = 0.04) are commonly associated with PVHI [12]. Plus, infection, prothrombotic disorders and dehydration are risk factors associated with CSVT [13, 14].

Therefore, we conducted a systematic review to explore the epidemiology and pathogenesis of PVHI; PAIS; and CSVT in preterm infants. To our knowledge, this is the first comprehensive systematic review of these three types of stroke in preterm infants.

## 2 Materials and methods

### 2.1 Literature search and selection

A systematic search was conducted from January 2000 to December 2019 using MEDLINE, EMBASE, SCOPUS and WEB OF SCIENCE (Science and Social Science Citation Index) databases based on the Cochrane recommendations for conducting reviews. Search terms included (perinatal stroke^*^) or (cerebral stroke^*^) or (neonatal stroke^*^) and (preterm^*^ or < 37 weeks^*^), as well as MeSH (Medical Subject Headings) (risk factor or etiology or outcome) with limits of English language and human studies applied. In addition, the reference list of the included studies for further relevant articles was hand-searched. The findings were reported according to the Meta-analyses Of Observational Studies in Epidemiology (MOOSE) checklist [15].

Studies were included in the systematic review if PVHI, PAIS and CSVT in preterm infants met the following criteria: (1) All original group study designs or case series; (2) studies published from January 2000 to December 2019 and (3) studies published in English. The exclusion criteria were: (1) studies where analysis of stroke in preterm and term infants could not be differentiated; (2) single case reports; (3) review papers and (4) abstracts and conference abstracts. If data were duplicated in > 1 study, then we included the study with the largest number of stroke cases.

### 2.2 Data extraction

An a priori designed data extraction tool based on the Cochrane recommendations was used. The following data were extracted from each study: First author’s last name, publication year, country where the study was performed, study design, study period, type of stroke, gestational age, birth weight, risk factors of preterm stroke, clinical presentation and outcome of each of the stroke types, as well as unadjusted and adjusted odds ratios, if reported. The quality of included studies were assessed using the Oxford centre for evidence-based medicine 2011 levels of evidence [16]. Study selection and data extraction were conducted independently by two investigators (BR and IN or MFE), with disagreements resolved by consensus.

### 2.3 Statistical analysis

All the findings were summarised and descriptive statistics used to describe the etiological and clinical variables extracted from papers. If the included studies reported on an individual case basis, proportions of the clinical variables were evaluated across the three stroke categories. A meta-analysis could not be conducted due to the heterogeneity of the reported data in the selected studies; explicitly, mismatched aims of the studies, discordant outcomes of stroke, and the small number stroke cases in each of the sub-groups for analysis. The statistician author (CG) deemed the conduct of any inferential analyses to be inappropriate. Associations were investigated for case by case where data were available. All analyses were conducted using STATA V.14 (STATA, Version 14, Stata Corp, College Station, TX, USA).

## 3 Results

### 3.1 Study characteristics

**Fig. 1:**
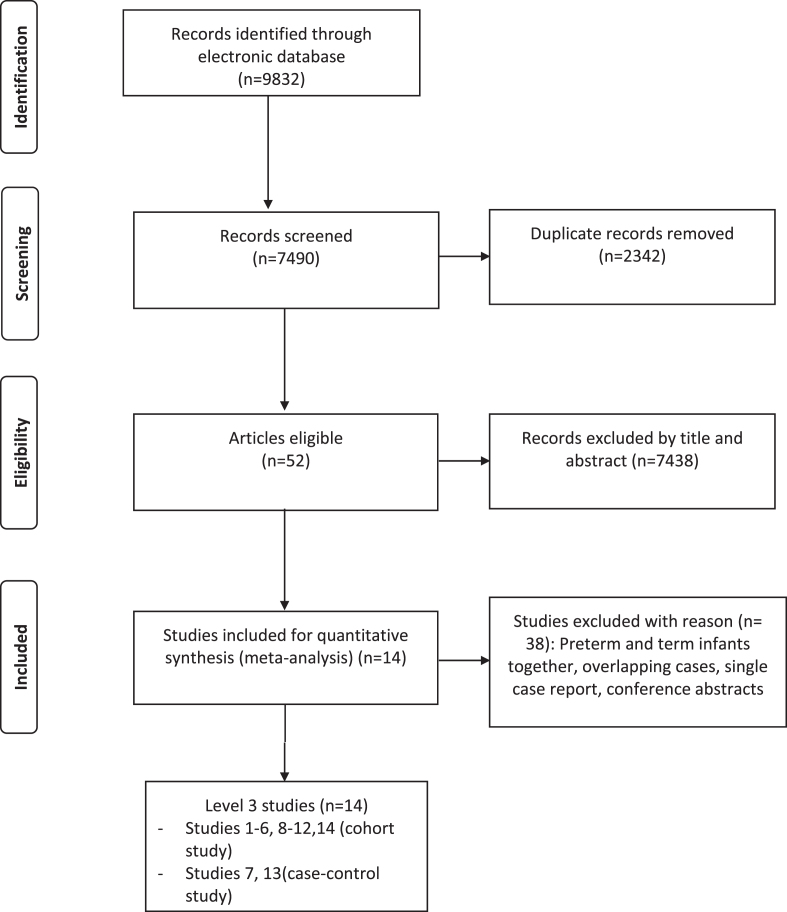
Flow chart of study selection using the PRISMA diagram [40].

The search strategy is summarised in the PRISMA flow diagram (Fig. 1). 7490 studies were identified and after removing the duplicates and examining the titles and abstracts, 52 studies were eligible for full-text review. Of these, 14 studies met the predetermined criteria and were included (Table 1). All the included studies were from high income countries. The studies were analysed based on the type of stroke (Table 1): PVHI [17–23], PAIS [24–28] and CSVT [29, 30] in the chronology of the publication year (starting with the most recent study).

**Table 1:** Characteristics of studies included in the systematic review

Study no:	Author (year)	Stroke type	Study origin	Study design	Study period	*n*	GA (weeks)	BW (grams)	OCEBM 2011 Levels of evidence [16]
Study 1	Roze et al. (2015) [17]	PVHI	Netherlands	Retrospective Cohort study	2007–2012	23	25–34	650–1950	Level 3
Study 2	Soltirovska Salamon et al. (2014) [18]	PVHI	Netherlands	Retrospective Cohort study	1990–2012	213	28–34	910–2965	Level 3
Study 3	Tsuji et al. (2014) [19]	PVHI	Japan	Retrospective Cohort study	1997–2005	22	23–31	538–1240	Level 3
Study 4	Harteman et al. (2012) [20]	PVHI	Netherlands	Retrospective Cohort study	2005–2010	62	25–34	580–2210	Level 3
Study 5	Roze et al. (2009) [21]	PVHI	Netherlands	Prospective Cohort study	1995–2003	38	25–35	700–2430	Level 3
Study 6	Dudink et al. (2008) [22]	PVHI	Netherlands	Retrospective Cohort study	2000 –2005	20	24–34	775–2800	Level 3
Study 7	Bassan et al. (2006) [23]	PVHI	USA	Retrospective Case-control study	1997 –2002	58	23–33	450–2375	Level 3
Study 8	Portale et al. (2019) [24]	PAIS	Italy	Retrospective Cohort study	January 1996–February 2015	2	35–36	3360–3500	Level 3
Study 9	Van der Aa et al. (2016) [25]	PAIS	Netherlands	Retrospective Cohort study	1990–2015	12	28–35	NA	Level 3
Study 10	Ecury-Goossen	PAIS	Belgium	Retrospective	1999–2011	25	24–36	NA	Level 3
et al. (2013) [26]	Cohort study
Study 11	Benders et al. (2009) [27]	PAIS	Netherlands	Prospective Cohort study	1990–2005	31	27–36	NA	Level 3
Study 12	Golomb et al. (2008) [28]	PAIS	USA	Retrospective Cohort study	1989–2006	23	23–35	535–2786	Level 3
Study 13	Raets et al. (2013) [29]	CSVT	Netherlands	Prospective Cross-control study	2009–2013	11	24–28	615–1185	Level 3
Study 14	Kersbergen et al. (2011) [30]	CSVT	Netherlands	Retrospective Cohort study	2002–2010	6	30–35	NA	Level 3
					2006–2009
Total	2000–2009 = 5 studies	PVHI = 7 studies		Prospective = 3 studies	1989 –2015	2000–2009 = 170	23–36	450–3500	Level 3 = 14
	2010–2020 = 9 studies	PAIS = 5 studies		Retrospective = 11 studies		2010–2020 = 376	weeks	grams	studies
		CSVT = 2 studies				Total = 546

BW –birth weight, CSVT –cerebral sinovenous thrombosis, GA –gestational age, n –population number, NA –not available, PAIS –perinatal arterial ischemic stroke, PVHI –periventricular hemorrhagic stroke. Oxford Centre for Evidence-based Medicine (OCEBM) –Level 3 (studies 1–14): ‘Local non-random sample’.

The eligible studies published between 2000 and 2019 included a total of 546 stroke cases from the period 1989 to 2015. The gestational ages and birth weights of the preterm infants were from 23–36 weeks and 450–3500 grams respectively.

### 3.2 Types of preterm strokes

#### 3.2.1 Periventricular hemorrhagic infarction

Eighty percent (*n* = 436/546) of the stroke cases in preterm infants resulted in PVHI. These infants were between 23–35 weeks of gestation and birth weights of 450–2965 grams. The studies included only unilateral PVHI cases. All 436 cases were diagnosed using cranial ultrasound (cUS). In addition, 21%(90/436) of the cases had brain magnetic resonance imaging (MRI) and 26%(23/90) had Diffusion Tensor Imaging (DTI) to predict the likelihood of unilateral spastic cerebral palsy (USCP) [17, 18, 20]. Serial EEG was conducted in 5%(23/436) to differentiate PVHI from PVL. Brain injury timing was determined using a combination of cUS and EEG to be post-natal in PVHI, but in utero in PVL [19].

The potential risk factors put forward in the literature for PVHI were genetic mutations of methylenetetrahydrofolate reductase (MTHFR) gene 88%, factor V Leiden gene 41%, COL4A1 gene 12%and prothrombin gene 6%[20]. However, many of these propositions have been disproven, specifically MTHFR [31] and more generally the minimal association between neonatal stroke and thrombophilia [32]. Some of the common clinical associations were emergency caesarean section in 30%, patent ductus arteriosus 19%, thrombocytopenia 18%, pneumothorax 12%, seizures 11%, intrauterine growth restriction and/or pre-eclampsia 8%[17–19, 21–23].

Studies [19, 22] focused on different factors to predict the outcomes of PVHI (e.g., anatomical location of brain injury versus timing of the injury versus types of cerebral veins involved), which precluded aggregation of findings across studies. Soltirovska Salamon et al. [18] (*n* = 213) classified PVHI into anatomical subtypes based on the location of the lesion: Parietal PVHI (84%), frontal PVHI (10%) and temporal PVHI (6%). This study was the only study to report the overall mortality of 33%based on anatomical subtypes, which included 99%for parietal PVHI and 1%for temporal PVHI. Thirty six percent of the infants with PVHI developed cerebral palsy (CP) and 75%of these had USCP. Ninety eight percent of the infants with CP had a parietal PVHI and only 2%had a temporal PVHI. Infants in Soltirovska Salamon et al. study [18] with temporal or frontal PVHI were deemed to be at higher risk for long-term cognitive, behavioural and visual problems. Harteman et al. [20] (*n* = 62) was the only study to classify PVHI based on the timing of the lesion, which was centred on their unit’s protocol for performing cUS. Typical PVHI (73%) was described when the onset of PVHI was within 6 –96 hours of age whereas atypical PVHI (27%) was when the onset of PVHI was < 6 hours or > 96 hours of age. Thrombophilia due to factor V Leiden mutation was more commonly associated with atypical PVHI. This study predicted higher prevalence of CP with atypical PVHI; 85%versus 12%with atypical and typical PVHI respectively.

Dudink et al. [22] (*n* = 20) classified PVHI into venous subtypes based on the types of cerebral vein affected. This study predicted 45%prevalence of CP in the cases with PVHI involving the terminal veins and normal outcome with involvement of the veins of the temporal and caudate area.

#### 3.2.2 Perinatal arterial ischemic stroke

Seventeen percent (*n* = 93/546) of the preterm infants included in this review were diagnosed with PAIS. These infants were between 23–36 weeks of gestation with birth weights of 535–3500 grams. More than 50%were middle cerebral artery (MCA) stroke; including left MCA and lenticulostriate branch [25, 27, 28]. The other types of PAIS were posterior cerebral artery (PCA) stroke, cerebellar infarcts involving posterior inferior cerebellar arteries and perforator stroke [26, 28]. All 93 cases were diagnosed by MRI.

Multivariate analysis by Benders et al. [27] (*n* = 31) indicated hypoglycemia, fetal hart rate abnormality and twin-to-twin transfusion syndrome as independent risk factors. Common clinical presentation was apnoea (83%) whilst seizures was an uncommon presentation amongst the preterm infants (10–30%) [27, 28]. The outcome of PAIS was dependent on gestational age; cortical sparing was noted in preterm infants compared to term infants (58%versus 4%) [25]. Golomb et al. [28] reported disability/neurological abnormality in 100%(22/22) and CP in 77%(17/22) of the surviving infants; 52%(12/23) had MCA stroke. Van der Aa et al. [25], Ecury-Goossen et al. [26] and Benders et al. [27] reported cumulative outcomes for preterm and term infants; impeding separate outcome analysis in the preterm infants.

#### 3.2.3 Cerebral sinovenous thrombosis

Three percent (17/546) of the stroke cases in preterm infants were CSVT [29, 30]. Theses infants were between 24–35 weeks of gestation. The study by Raets et al. [29] was a prospective case control study (*n* = 11 with CSVT versus *n* = 238 without CSVT) of preterm infants < 29 weeks of gestation. All 11 cases had partial or complete thrombosis in the transverse sinus, including one case with multiple sinus thrombosis. All the cases were diagnosed by cUS, although 64%(7/11) were confirmed by MRI. None of the potential risk factors such as sepsis, patent ductus arteriosus and Apgar scores were significantly different between the CSVT and non-CSVT groups. The outcomes at 6 months to 2 years included: One neonatal death; 50%(5/10) were typically developing and 50%had abnormal Bayley scales of infant development or mental or psychomotor developmental assessment scores suggesting long-term disability.

The study by Kersbergen et al. [30] was a retrospective cohort study (*n* = 6 preterm infants and *n* = 24 full term infants) of preterm infants≥30 weeks of gestation. In this study, all six cases had straight sinus thrombosis and 83%(5/6) had multiple sinus thrombosis and only 33%(2/6) had transverse sinus thrombosis. The preterm infants had severe white matter lesions (*p* < 0.001) in contrast to the term infants who had predominant punctate white matter lesions (*p* < 0.001). All six preterm infants were diagnosed using MRI or magnetic resonance venography (MRV). Outcomes in these cases at 3 years were: 83%(5/6) mortality and in the one surviving child CP with epilepsy.

## 4 Discussion

Stroke in infants is an important cause of CP and other neurological disabilities [33]. Strategies to improve the overall outcome of preterm infants should include timely diagnosis of stroke, which may be challenging due to the coexisting morbidities of prematurity that confound identification of stroke [34]. This systematic review confirms the paucity of epidemiologic data and outcome data for the three most common types of stroke in preterm infants; namely, PVHI, PAIS and CSVT. In this review, approximately 70%of the stroke cases were reported in the second epoch of publications (2010 –2019) compared to 30%in the previous decade. This may be due to increased awareness of stroke in this population and the emergence of more sophisticated neuroimaging techniques such as brain MRI with DTI, magnetic resonance angiography (MRA) and MRV coupled with the routine use of cUS [35].

The three types of stroke in preterm infants included in this review were PVHI, PAIS and CSVT. Different studies used different techniques to classify PVHI to accurately predict the long-term outcome based on the anatomical location of the PVHI, type of cerebral venous involvement and on the timing of the occurrence of PVHI [18, 20, 22]. Parietal PVHI was more common than temporal and frontal lobe PVHI [18].

Data regarding PAIS were scarce in preterm infants [27, 28, 36]. Left MCA stroke was most common compared to right MCA, PCA, and cerebellar strokes [27, 28]. For infants between 28–32 weeks’ gestation, lenticulostriate branch of the MCA was most often involved. Whereas, for infants > 32 weeks’ gestation, main branches of the MCA were more often involved [27]. Perforator stroke was under-recognised because of the lack of clinical symptoms [26]. CSVT was the least reported type of preterm stroke in the literature [13, 37].

A clear understanding of the mechanism and identification of specific risk factors may aid in devising future novel preventative strategies for preterm stroke. PVHI and CSVT are due to obstruction in the venous drainage following germinal matrix hemorrhage and blockage of a dural sinus by thrombus respectively. PAIS is secondary to lack of blood supply following a thrombus in the cerebral artery. The potential risk factors were hemodynamic instability, cerebral blood flow abnormalities and genetic triggers or septicemia causing thrombophilia [20, 23, 24, 27]. The factors associated with flow abnormalities in the fetal brain were emergency caesarean section [18–20], fetal distress/low Apgar scores/resuscitation [23] and pneumothorax [21].

cUS with doppler technology was widely used to diagnose PVHI MRI with diffusion-weighted imaging (DWI) sequences and MRV is the gold standard [38, 39]. Diffusion tensor imaging (DTI) identified the microstructural properties of the white matter in the posterior limb of the internal capsule, elucidating the risk for motor impairment [17]. Asymmetry visible on the DTI was a reliable predictor of the development of USCP [17].

Early diagnosis of stroke assists in the recovery process through neuroplasticity though the scope of healing also depends on the gestational age vis-á-vis the embryological development of the preterm brain. Parietal PVHI had a poorer prognosis with 40%mortality rate and 50%risk of CP, while, temporal PVHI and frontal PVHI had increased risk of cognitive, behavioural and visual problems [18, 22].

Amalgamated reporting of stroke in preterm and term infants was the major limitation of this review, preventing disaggregation of data across studies into a meta-analysis. There was also a potential risk of duplication of cases because of multiple reporting of the same stroke cases across some studies. The results should be interpreted cautiously because the individual case data were often reported retrospectively and as a secondary end-point. The review also included a heterogenous studies eg not same aims of the studies (aimed for etiology, clinical presentation, investigations and or outcome of stroke) and not same outcomes of stroke (outcomes at 6 months to 3 years and anytime in between).

Stroke in preterm infants is unlikely to present with focal seizures and may be asymptomatic or have nonspecific clinical presentation. Therefore, appropriate neuroimaging cUS in presence of three or more risk factors of stroke should become part of routine screening in all preterm babies. In addition, analysis of stroke in preterm and term infants separately is likely to yield a clearer understanding of the pathophysiology unique to preterm infants.

## 5 Conclusion

In conclusion, this systematic review confirms, that, the etiology of stroke in preterm infants is not known and the precise causal pathway is not always very well understood and more research is required. The outcome of stroke was likely to be complicated by the co-morbidities of prematurity, though, it was outside the scope of this review. Early identification and the reported prevalence rate of stroke may increase with greater recognition of the clinical presentation of stroke, routine cUS and brain MRI in high-risk preterm groups.

## Sources of funding

No funding was secured and there were no financial relationships to disclose.

## Disclosures

None.

## Contributors’ statement

Dr Bithi Roy conceptualized and designed the study, designed data collection instruments (first extractor), carried out the initial analyses, drafted, reviewed and revised the manuscript. Prof Iona Novak assisted with data extraction (additional extractor), analyses, critically reviewed the manuscript for important intellectual content and revised the manuscript. Dr Megan Finch-Edmondson assisted with data extraction (additional extractor), reviewed and revised the manuscript. Claire Galea assisted with statistical analysis, reviewed and revised the manuscript. Dr Catherine Morgan, A/Prof Karen Walker and Prof Nadia Badawi critically reviewed and revised the manuscript for important intellectual content. All authors approved the final manuscript as submitted and agree to be accountable for all aspects of the work.

## References

[1] Engle WA , American academy of pediatrics committee on fetus and newborn. Age terminology during the perinatal period. Pediatrics. 2004;114(5):1362–4. 15520122 10.1542/peds.2004-1915

[2] Kirton A , Deveber G . Life after perinatal stroke. Stroke. 2013;44(11):3265–71. 24105698 10.1161/STROKEAHA.113.000739

[3] Dunbar M , Kirton A . Perinatal stroke: Mechanisms, management, and outcomes of early cerebrovascular brain injury. Lancet Child Adolesc Health. 2018;2(9):666–76. 30119760 10.1016/S2352-4642(18)30173-1

[4] Collaborators GBDS. Global, regional, and national burden of stroke, 1990-2016: A systematic analysis for the Global Burden of Disease Study 2016. Lancet Neurol. 2019;18(5):439–58. 30871944 10.1016/S1474-4422(19)30034-1 PMC6494974

[5] Dunbar M , Mineyko A , Hill M , Hodge J , Floer A , Kirton A . Population Based Birth Prevalence of Disease-Specific Perinatal Stroke. Pediatrics. 2020;146(5):11. 10.1542/peds.2020-013201 33115795

[6] Laugesaar R , Kolk A , Tomberg T , Metsvaht T , Lintrop M , Varendi H , et al. Acutely and retrospectively diagnosed perinatal stroke: A population-based study. Stroke. 2007;38(8):2234–40. 17585082 10.1161/STROKEAHA.107.483743

[7] Chabrier S , Husson B , Dinomais M , Landrieu P , Nguyen The Tich S . New insights (and new interrogations) in perinatal arterial ischemic stroke. Thromb Res. 2011;127(1):13–22. 21055794 10.1016/j.thromres.2010.10.003

[8] Medley TL , Miteff C , Andrews I , Ware T , Cheung M , Monagle P , et al. Australian clinical consensus guideline: The diagnosis and acute management of childhood stroke. Int J Stroke. 2019;14(1):94–106. 30284961 10.1177/1747493018799958

[9] Volpe JJ . Brain injury in the premature infant–from pathogenesis to prevention. Brain Dev. 1997;19(8):519–34. 9440796 10.1016/s0387-7604(97)00078-8

[10] Benders MJ , Groenendaal F , Uiterwaal CS , Nikkels PG , Bruinse HW , Nievelstein RA , et al. Maternal and infant characteristics associated with perinatal arterial stroke in the preterm infant. Stroke. 2007;38(6):1759–65. 17495219 10.1161/STROKEAHA.106.479311

[11] Lee J , Croen LA , Backstrand KH , Yoshida CK , Henning LH , Lindan C , et al. Maternal and infant characteristics associated with perinatal arterial stroke in the infant. JAMA. 2005;293(6):723–9. 15701914 10.1001/jama.293.6.723

[12] Harteman JC , Nikkels PG , Kwee A , Groenendaal F , de Vries LS . Patterns of placental pathology in preterm infants with a periventricular haemorrhagic infarction: Association with time of onset and clinical presentation. Placenta. 2012;33(10):839–44. 22835681 10.1016/j.placenta.2012.06.014

[13] deVeber G , Andrew M , Adams C , Bjornson B , Booth F , Buckley DJ , et al. Cerebral sinovenous thrombosis in children. N Engl J Med. 2001;345(6):417–23. 11496852 10.1056/NEJM200108093450604

[14] Javed I , Sultan T , Rehman ZU , Yaseen MR . Clinical spectrum and outcome of cerebral venous sinus thrombosis in children. J Coll Physicians Surg Pak. 2018;28(5):390–3. 29690971 10.29271/jcpsp.2018.05.390

[15] Stroup DF , Berlin JA , Morton SC , Olkin I , Williamson GD , Rennie D , et al. Meta-analysis of observational studies in epidemiology: A proposal for reporting. Meta-analysis Of Observational Studies in Epidemiology (MOOSE) group. JAMA. 2000;283(15):2008–12. 10789670 10.1001/jama.283.15.2008

[16] OCEBM levels of Evidence Working group. The Oxford 2011 levels of evidence. 2011.

[17] Roze E , Benders MJ , Kersbergen KJ , van der Aa NE , Groenendaal F , van Haastert IC , et al. Neonatal DTI early after birth predicts motor outcome in preterm infants with periventricular hemorrhagic infarction. Pediatr Res. 2015;78(3):298–303. 25978802 10.1038/pr.2015.94

[18] Soltirovska Salamon A , Groenendaal F , van Haastert IC , Rademaker KJ , Benders MJNL , Koopman C , et al. Neuroimaging and neurodevelopmental outcome of preterm infants with a periventricular haemorrhagic infarction located in the temporal or frontal lobe. Dev Med Child Neurol. 2014;56(6):547–55. 24506484 10.1111/dmcn.12393

[19] Tsuji T , Okumura A , Kidokoro H , Hayakawa F , Kubota T , Maruyama K , et al. Differences between periventricular hemorrhagic infarction and periventricular leukomalacia. Brain Dev. 2014;36(7):555–62. 23978489 10.1016/j.braindev.2013.07.014

[20] Harteman JC , Groenendaal F , van Haastert IC , Liem KD , Stroink H , Bierings MB , et al. Atypical timing and presentation of periventricular haemorrhagic infarction in preterm infants: the role of thrombophilia. Dev Med Child Neurol. 2012;54(2):140–7. 22098125 10.1111/j.1469-8749.2011.04135.x

[21] Roze E , Van Braeckel KN , van der Veere CN , Maathuis CG , Martijn A , Bos AF . Functional outcome at school age of preterm infants with periventricular hemorrhagic infarction. Pediatrics. 2009;123(6):1493–500. 19482759 10.1542/peds.2008-1919

[22] Dudink J , Lequin M , Weisglas-Kuperus N , Conneman N , Van Goudoever JB , Govaert P . Venous subtypes of preterm periventricular haemorrhagic infarction. Arch Dis Child Fetal Neonatal Ed. 2008;93(3):F201–F6. 17768152 10.1136/adc.2007.118067

[23] Bassan H , Feldman HA , Limperopoulos C , Benson CB , Ringer SA , Veracruz E , et al. Periventricular hemorrhagic infarction: risk factors and neonatal outcome. Pediatr Neurol. 2006;35(2):85–92. 16876002 10.1016/j.pediatrneurol.2006.03.005

[24] Portale A , Fiumara A , Scalora L , Greco F , Smilari P , Venti V , et al. Arterial ischemic stroke (AIS) in childhood: Clinical report from a single control center. Childs Nerv Syst. 2019;35(2):283–93. 30542811 10.1007/s00381-018-4017-1

[25] van der Aa NE , Benders MJ , Nikkels PG , Groenendaal F , de Vries LS . cortical sparing in preterm ischemic arterial stroke. Stroke. 2016;47(3):869–71. 26757751 10.1161/STROKEAHA.115.011605

[26] Ecury-Goossen GM , Raets MM , Lequin M , Feijen-Roon M , Govaert P , Dudink J . Risk factors, clinical presentation, and neuroimaging findings of neonatal perforator stroke. Stroke. 2013;44(8):2115–20. 23723310 10.1161/STROKEAHA.113.001064

[27] Benders MJ , Groenendaal F , De Vries LS . Preterm arterial ischemic stroke. Semin Fetal Neonatal Med. 2009;14(5):272–7. 19656748 10.1016/j.siny.2009.07.002

[28] Golomb MR , Garg BP , Edwards-Brown M , Williams LS . Very early arterial ischemic stroke in premature infants. Pediatr Neurol. 2008;38(5):329–34. 18410848 10.1016/j.pediatrneurol.2007.12.012 PMC2770811

[29] Raets MM , Sol JJ , Govaert P , Lequin MH , Reiss IK , Kroon AA , et al. Serial cranial US for detection of cerebral sinovenous thrombosis in preterm infants. Radiology. 2013;269(3):879–86. 23985276 10.1148/radiol.13130401

[30] Kersbergen KJ , Groenendaal F , Benders MJ , van Straaten HL , Niwa T , Nievelstein RA , et al. The spectrum of associated brain lesions in cerebral sinovenous thrombosis: relation to gestational age and outcome. Arch Dis Child Fetal Neonatal Ed.F. 2011;96(6):404–9. 10.1136/adc.2010.201129 21317440

[31] Hickey SE , Curry CJ , Toriello HV . ACMG practice guideline: Lack of evidence for MTHFR polymorphism testing. Genet Med. 2013;15(2):153–6. 23288205 10.1038/gim.2012.165

[32] Curtis C , Mineyko A , Massicotte P , Leaker M , Jiang XY , Floer A , et al. Thrombophilia risk is not increased in children after perinatal stroke. Blood. 2017;129(20):2793–800. 28258054 10.1182/blood-2016-11-750893

[33] Greenham M , Gordon A , Anderson V , Mackay MT . Outcome in childhood stroke. Stroke. 2016;47(4):1159–64. 26956257 10.1161/STROKEAHA.115.011622

[34] Badawi N , McIntyre S , Hunt RW . Perinatal care with a view to preventing cerebral palsy. Dev Med Child Neurol. 2021;63(2):156–61. 33251607 10.1111/dmcn.14754 PMC7839537

[35] Kirton A , de Veber G . Stroke in the fetus and neonate. Future Cardiol. 2006;2(5):593–604. 19804197 10.2217/14796678.2.5.593

[36] Benders MJ , Groenendaal F , Uiterwaal CS , de Vries LS . Perinatal arterial stroke in the preterm infant. Semin Perinatol. 2008;32(5):344–9. 18929157 10.1053/j.semperi.2008.07.003

[37] Berfelo FJ , Kersbergen KJ , van Ommen CH , Govaert P , van Straaten HL , Poll-The BT , et al. Neonatal cerebral sinovenous thrombosis from symptom to outcome. Stroke. 2010;41(7):1382–8. 20522810 10.1161/STROKEAHA.110.583542

[38] Dudink J , Mercuri E , Al-Nakib L , Govaert P , Counsell SJ , Rutherford MA , et al. Evolution of unilateral perinatal arterial ischemic stroke on conventional and diffusion-weighted MR imaging. AJNR Am J Neuroradiol. 2009;30(5):998–1004. 19193752 10.3174/ajnr.A1480 PMC7051645

[39] Kuker W , Mohrle S , Mader I , Schoning M , Nagele T . MRI for the management of neonatal cerebral infarctions: importance of timing. Childs Nerv Syst. 2004;20(10):742–8. 15490191 10.1007/s00381-004-0988-1

[40] PRISMA. PRISMA. PRISMA 2009 checklist 2009.

